# General somatic health and lifestyle habits in individuals with obsessive- compulsive disorder: an international survey

**DOI:** 10.1186/s12888-024-05566-w

**Published:** 2024-02-05

**Authors:** Anna Holmberg, Lina Martinsson, Matthias Lidin, Christian Rück, David Mataix-Cols, Lorena Fernández de la Cruz

**Affiliations:** 1 Centre for Psychiatry Research, Department of Clinical Neuroscience, Karolinska Institutet, Gävlegatan 22B, 8th floor, Stockholm, 113 30 Sweden; 2 Stockholm Health Care Services, Region Stockholm, Stockholm, Sweden; 3 Department of Medicine, Karolinska Institutet, Stockholm, Sweden; 4 Department of Cardiology, Heart, Vascular and Neuro Theme, Karolinska University Hospital, Stockholm, Sweden; 5 Department of Clinical Sciences, Lund University, Lund, Sweden

**Keywords:** Obsessive-compulsive disorder, Physical health, Cardiometabolic risk factors, Lifestyle habits, Physical activity, Dietary habits, Alcohol consumption, Drug consumption, Sleep, Prevention

## Abstract

**Background:**

Obsessive-compulsive disorder (OCD) has been associated with a broad range of health-related issues. Unhealthy lifestyle habits such as physical inactivity, an unhealthy diet, smoking, and alcohol consumption are hypothesized to contribute to this association. However, the lifestyle habits of individuals with OCD have been scarcely investigated. In this international survey, we explored the physical health and lifestyle habits of adults with a self-reported diagnosis of OCD.

**Methods:**

An online global survey available in seven languages was disseminated through interest organizations and social media between July 2021 and March 2022. The survey included questions relating to socio-demographic variables and clinical characteristics (including OCD symptom severity – as measured with the 12-item self-report scale Obsessive-Compulsive Inventory [OCI-12] – and psychotropic medication), physical health, and lifestyle habits. Frequencies and percentages, or means and standard deviations, as appropriate, were calculated. Subgroup analyses by OCD symptom severity, gender, and age group were performed.

**Results:**

A total of 496 individuals with OCD completed the survey and were included in the analyses (mean age = 36.0 years, *SD* = 12.5, range 18–79; 78.8% women). Most participants were from Europe (*n =* 245, 49.4%) and North America (*n =* 187, 37.7%). OCD symptom severity scores were on the moderate range (OCI-12 mean score = 21.2, *SD* = 9.1). A majority (*n =* 354, 71.4%) reported having comorbid somatic health issues, mainly allergies, gastrointestinal conditions, and cardiometabolic conditions. Nearly half of the sample (*n =* 236, 47.6%) reported a body mass index ≥ 25, corresponding to at least overweight. A significant proportion of the participants reported low physical activity (*n =* 271, 55.0%), unhealthy dietary habits (*n =* 182, 36.7%), risk consumption of alcohol (*n =* 111, 22.3%), and non-restorative sleep (*n =* 268, 54.0%). Subgroup analyses showed overall similar results across groups, with some exceptions.

**Conclusions:**

In this sample, individuals with OCD self-reported a range of health-related issues and a number of unhealthy lifestyle behaviors, most prominently a lack of physical activity. Interventions aimed at modifying unhealthy lifestyles to prevent or improve health conditions beyond the psychiatric symptoms should be considered.

**Supplementary Information:**

The online version contains supplementary material available at 10.1186/s12888-024-05566-w.

## Background

Obsessive-compulsive disorder (OCD) is a common and debilitating mental health disorder affecting around 1–2% of the population [1, 2]. The disorder is associated with decreased quality of life and a range of psychiatric comorbidities [1].

Similar to other mental health conditions [3–5], OCD has been associated with increased risk of premature death due to both natural and unnatural causes [6, 7] and increased morbidity rates [8]. For example, large epidemiological studies have shown strong associations between OCD and autoimmune diseases [9–11]. Additionally, individuals with OCD in specialist settings have shown a higher prevalence of metabolic syndrome, compared with the general population [12, 13]. A nationwide epidemiological study in Sweden also showed that individuals with OCD have an increased risk of cardiometabolic complications, including obesity, dyslipidemia, type 2 diabetes mellitus, and a range of circulatory system diseases, compared to individuals without a diagnosis of OCD [14]. Individuals with OCD also have a moderately increased risk of cardiovascular diseases, compared to matched controls without the disorder and their siblings [15]. The observed risks of cardiometabolic disorders seem to be at least partially independent from unmeasured familial confounding and the use of psychotropic medication [14, 15]. There is also preliminary evidence of an association between OCD and respiratory diseases [16, 17], gastrointestinal diseases [13, 16], insomnia [18, 19], migraine [17, 20], dementia [21], and chronic pain [16, 22].

Unhealthy lifestyle habits such as physical inactivity, an unhealthy diet, smoking, and alcohol consumption are hypothesized to contribute to somatic morbidity [14, 15, 23]. Most importantly, these modifiable lifestyle habits have been highlighted as key factors to tackle to protect physical health in people with mental illness [5]. However, lifestyle factors have barely been studied in individuals with OCD and, currently, it is unclear whether individuals with OCD have unhealthier lifestyles than the general population. Two studies in clinical samples have described a lack of physical activity in individuals with OCD, especially in those with metabolic syndrome or other medical conditions [12, 13]. To our knowledge, only one study has so far investigated overall dietary quality in individuals with OCD. The dietary patterns and nutrient intake of 85 individuals with OCD from Australia were evaluated using a brief questionnaire, showing that the dietary recommendations were largely followed [24]. However, given that this study included a relatively small number of individuals and relied on self-reported information, the dietary habits of individuals with OCD deserve further examination in larger samples, different settings, and using different methods. Meta-analytic evidence from 67 publications [25] concluded that about 27% of individuals with OCD are likely to smoke, compared to the 15% estimated in the general population [26]. Nonetheless, the prevalence of smoking has decreased over time and varies by country, with recent estimations being 5.8% in Sweden [27] or 12.5% in the United States [28]. The evidence regarding the association between OCD and substance use disorders is more mixed [29–31]. However, a recent nationwide register study concluded that individuals with OCD had a significantly increased risk of alcohol-related and other drug-related disorders [32]. Nevertheless, it is important to acknowledge that hazardous use of alcohol or drugs and substance use disorders are overlapping but different constructs. Therefore, there will be individuals with OCD who do not meet criteria for substance use disorders but may still be classified as having a risky consumption of alcohol and drugs, which would also be important to identify and address. Further, sleep disturbances have been shown to be prevalent in individuals with OCD in terms of poor sleep quality, reduced sleep time, and higher prevalence of insomnia, compared to the general population [18, 19, 33–35].

Thus, while the evidence is of mixed quality, there are reasons to believe that the lifestyles of individuals with OCD may contribute to increased physical morbidity in this group. However, it is crucial to have a deeper understanding of the lifestyles of individuals with OCD and, importantly, how they vary according to clinical characteristics, such as symptom severity. This will allow for a more rational development of lifestyle modification interventions for individuals with OCD. Here, we report the results of an international survey of almost 500 individuals who self-reported a diagnosis of OCD collecting information on their clinical characteristics, somatic health, and lifestyle habits.

## Methods

### Participants and procedures

Individuals aged 18 years or older reporting a confirmed diagnosis of OCD were welcome to participate in the study. Recruitment was done via the Internet. Information about the study and a link to the survey were shared on social media (Twitter, Facebook, and Reddit) and in the websites, or via the members’ email distribution list, of several collaborating service user organizations in different countries.

Upon entering the survey, and prior to the start of the questions, potential participants had to confirm that they understood that their answers would be used for research purposes and that that they agreed to participate in the study. This constituted written consent. All answers were anonymous and no identifiable data were stored. The study was approved by the Swedish Ethical Review Authority (registration number 2021–02608).

### Survey design

An online survey (available as Supplementary material) was developed and placed on a widely used Internet platform (http://alchemer.com). In order to gather information on individuals from several countries, the survey was originally developed in English and translated into six additional languages, including Swedish, Dutch, Spanish, Catalan, Japanese, and Chinese, by members of the research group that were native speakers of these languages. The survey took about 10–15 min to complete. The content was divided into several sections, including both multiple choice and open-ended questions. Most of the questions were compulsory and skip logic was used when appropriate.

The first section gathered socio-demographic information (e.g., gender, age, country, education level, employment status) and clinical characteristics relating to mental health, including reporting of a confirmed diagnosis of OCD, self-reported psychiatric comorbidities, and previous or current psychological treatment for OCD or psychotropic medication.

A second section on physical health asked about weight and height in order to calculate the participants’ BMI (weight/height^2^). The World Health Organization (WHO) classification was used to group the BMI scores, by which a BMI < 18.5 was classified as underweight, 18.5 to 24.9 as normal weight, 25 to 29.9 as overweight, and ≥ 30 as obese [36]. The participants were also asked about presence of different somatic conditions, including previous acute cardiovascular or cerebrovascular event, cardiovascular disorder, hypertension, type 2 diabetes, and high cholesterol (also grouped together as cardiometabolic conditions), gastrointestinal conditions, respiratory conditions, allergies, musculoskeletal conditions, autoimmune diseases, neurological diseases, migraine headaches, cancer, and other health problems. Participants also reported on whether they were taking any medications for these conditions. Additionally, this section included the first question from the 36-item Short Form of Health Survey (SF-36) [37], which asks the responder to rate their health status in a 5-point Likert scale, from excellent to poor. Meta-analytical evidence has shown that answers to this type of single-item self-rated general health questions predict mortality risk [38].

The following section consisted of questions concerning lifestyle habits. *Physical activity* was measured with the International Physical Activity Questionnaire (IPAQ), short version [39]. The IPAQ is one of the most widely used physical activity questionnaires, consisting of 7 items concerning physical activity (walking, moderate intense activities, and vigorous intense activities) during the last 7 days. The scale was slightly modified to include fixed answer options, instead of free-text answers, to better suit the survey structure. The fixed answer options were designed to correspond to the three different physical activity categories presented in the scoring guidelines of the IPAQ protocol [40]. Participants responses were converted to averaged metabolic equivalent of task (MET) minutes, using the formula from the IPAQ scoring protocol [40]. The total MET minutes/week was calculated by adding up all physical activities. Participants were then divided into three different categories based on the criteria in the IPAQ scoring protocol. A total of 1500 MET minutes/week or more were categorized as high physical activity, 600 to 1500 MET minutes/week were considered moderate, and those with 600 or less MET minutes/week were included in the low physical activity category.

*Dietary habits* were assessed with five questions from a dietary questionnaire created by the Swedish National Board of Health and Welfare [41], which are based on the Nordic Nutrition Recommendations [42]. The addition of four of the questions in this questionnaire results into a dietary index score ranging from 0 to 12 points, where 9 to 12 points are classified as a healthy diet, 5 to 8 points as a relatively healthy diet, and 0 to 4 points as unhealthy dietary habits [41]. The fifth question, not included in the scoring, concerns breakfast habits, given that regular breakfast habits have shown to be associated with less overeating during the day, as well as increased physical activity [41, 43, 44]. Additional questions on consumption of meat and salt were also added, modified from the STEPS Instrument developed by the WHO [45] and the National Health and Nutrition Examination Survey in the United States [46]. Excessive consumption of meat and salt is linked to various health risks [47, 48], and their intake is advised to be restricted [42].

Current and previous *tobacco use* was recorded by asking purposely-created questions about whether consumption was present and number of cigarettes smoked per day or number of cans/boxes of snuff/smokeless tobacco consumed per week. *Alcohol consumption* was measured with the Alcohol Use Disorders Identification Test– Concise (AUDIT-C) [49], a brief screening tool to detect problematic drinking. Scores in the AUDIT-C range from 0 to 12 points, where 0 points indicate no alcohol use. Cut-off scores for risk consumption vary across settings and population [50], but a score ≥ 4 points has been suggested as a cut-off for detecting risk consumption of alcohol when validated in the general population [50, 51]. The AUDIT-C has shown acceptable psychometric properties [50]. *Drug use* was collected by asking one question concerning frequency of use of illicit substances during the last year validated by Smith and Schmidt [52]. The question included minor modifications in the wording and pre-determined answer options were added to match the options in the alcohol consumption section.

*Sleep habits* were evaluated by using two questions about the number of daily hours of sleep and, as a measure of sleep quality, how often they woke up feeling fresh and rested. This latter question was modified from the 5-item WHO Well-Being Index (WHO-5) [53], including slight wording modifications.

The participants were also asked whether they had considered changing their lifestyle habits and, if so, what they wanted to change. The following questions concerned previous lifestyle changes and reasons for being successful or not with changing their lifestyle habits.

Current obsessive-compulsive symptom severity was measured with the 12-item self-report scale Obsessive-Compulsive Inventory (OCI-12) [54]. The scale has shown good to excellent psychometric properties and good sensitivity and specificity for clinical cut-off scores. As per the original validation paper [54], scores between 0 and 12 indicated mild, between 13 and 21 indicated moderate, and between 22 and 48 indicated severe OCD symptoms.

### Statistical analyses

The completed surveys were downloaded into a spreadsheet. Quantitative statistical analyses were performed using Stata 16.1 (StataCorp LLC). Frequencies and percentages for each question were calculated. Survey answers were compared across a number of different categorical variables, including OCD severity (divided into mild, moderate, and severe, according to the severity cut-off scores of the OCI-12) [54], gender (woman, man, other/prefer not to say), and age group (18–30 years, 31–45 years, 46 and older) by using Chi-square tests of independence, for categorical variables, followed by subsequent 2 × 2 Chi-square tests to examine differences between groups, if relevant [55], or one-way analysis of the variance (ANOVA), for continuous variables, followed by post-hoc pairwise comparisons using Tukey (in cases of equal variances) or Tamhane’s T2 (in cases of unequal variances), if relevant. The statistical significance threshold was set at *p* < 0.05.

## Results

### Demographic and clinical characteristics

Data were collected from July 2021 to March 2022. A total of 641 individuals completed the survey, and 144 were excluded because they did not fulfill the inclusion criteria (139 did not have a formal OCD diagnosis and 5 were younger than 18). One additional participant was excluded due to a stereotypic response pattern and incongruent answers. Hence, the final sample included 496 individuals.

Demographic and clinical characteristics of the final sample are shown in Table 1. The majority of the participants were women (*n* = 391, 78.8%) and their mean age was 36.0 years (*SD* = 12.5, range 18–79). Participants were from 26 different countries, most of them located in Europe (*n* = 245, 49.4%) and North America (*n* = 187, 37.7%).

**Table 1:** Demographic and clinical characteristics of the survey participants (*N* = 496)

Variable	*n* (%)
**Gender**	
Woman	391 (78.8)
Man	92 (18.6)
Other/prefer not to say	13 (2.6)
**Age**	
Mean *(SD)*	36.0 (12.5)
Median *(IQR)*	34 (18–70)
18–30 years	196 (39.5)
31–45 years	180 (36.3)
46 and older	120 (24.2)
**Country**	
United States	180 (36.3)
Sweden	142 (28.6)
Japan	53 (10.7)
Spain	52 (10.5)
United Kingdom	18 (3.6)
The Netherlands	18 (3.6)
Other countries	29 (6.7)
**Education (highest completed)**	
Less than primary school	3 (0.6)
Primary school completed	32 (6.5)
Secondary school completed	159 (32.1)
College/University	227 (45.8)
Post-graduate degree	75 (15.1)
**Occupation**	
Employed full-time	192 (38.7)
Employed part-time	64 (12.9)
Unemployed (able to work)	32 (6.5)
Unemployed (unable to work)	60 (12.1)
Retired	14 (2.8)
Student	76 (15.3)
Other	58 (11.7)
**Psychiatric comorbidities**	
Any	418 (84.3)
Anxiety disorder	270 (54.4)
Depression	265 (53.4)
Attention-deficit/hyperactivity disorder	72 (14.5)
Autism spectrum disorder	50 (10.1)
Body dysmorphic disorder	22 (4.4)
Hoarding disorder	14 (2.8)
Skin-picking disorder	55 (11.1)
Hair-pulling disorder (trichotillomania)	27 (5.4)
Eating disorder	69 (13.9)
Bipolar disorder	34 (6.9)
Psychosis	14 (2.8)
Substance use disorder	22 (4.4)
Other psychiatric disorder	54 (10.9)
**OCD severity**	
OCI-12 total score, Mean *(SD)*	21.2 (9.1)
Mild	106 (21.4)
Moderate	162 (32.7)
Severe	228 (45.9)

*Abbreviations: IQR*, Interquartile range; *OCI-12*, 12-item Obsessive-Compulsive Inventory; *SD*, standard deviation

The mean score on the OCI-12 for the whole sample was 21.2 (*SD* = 9.1), corresponding to moderate severity. According to the severity categories, 106 individuals (21.4%) were considered to have mild OCD symptom severity, 162 individuals (32.7%) were classified as moderate, and 228 individuals (45.9%) as severe. Mean age of onset of the OCD symptoms (reported by *n =* 483 individuals) was 16.0 years old (*SD* = 9.5). Psychiatric comorbidity was common, with 418 individuals (84.3%) reporting at least one psychiatric comorbidity, mainly anxiety disorders (*n* = 270, 54.4%) and depression (*n* = 265, 53.4%).

A large majority reported to have taken medication for their OCD at some point in their lifetime (*n =* 393, 79.2%), with 307 (61.9%) currently on medication. Of these, 260 (84.7%) were taking selective serotonin reuptake inhibitors (SSRIs). Another 392 (79.0%) of the total sample had received psychological treatment for their OCD at some point, and 200 (40.3%) were currently receiving it.

### Somatic health

The results of the somatic health questions are shown in Table 2. Full results on these outcomes by OCD symptom severity, gender, and age group are presented in Supplementary Tables 1–3 and key results are summarized below.

**Table 2:** Somatic health of the survey participants (*N* = 496)

Variable	*n* (%)
**Self-rated health**	
Excellent	21 (4.2)
Very good	102 (20.6)
Good	200 (40.3)
Fair	132 (26.6)
Poor	41 (8.3)
**Health problems**	
Any	354 (71.4)
Allergies	192 (38.7)
Gastrointestinal conditions	134 (27.0)
Cardiometabolic conditions	97 (19.6)
Hypertension	58 (11.7)
High cholesterol	52 (10.5)
Type 2 diabetes	18 (3.6)
Cardiovascular disorder	11 (2.2)
Previous acute cardiovascular or cerebrovascular event	2 (0.4)
Migraine headaches	83 (16.7)
Musculoskeletal conditions	74 (14.9)
Respiratory conditions	47 (9.5)
Autoimmune diseases	44 (8.9)
Neurological diseases	11 (2.2)
Cancer	9 (1.8)
Other health problem	89 (17.9)
**Body Mass Index**	
Mean *(SD)*	26.1 (6.5)
Obese	123 (24.8)
Overweight	113 (22.8)
Normal weight	227 (45.8)
Underweight	29 (5.9)
**Currently taking medication for somatic health issues**	195 (39.3)

*Abbreviation: SD*, standard deviation

About two thirds of the sample (*n* = 323, 65.1%) self-rated their health as excellent, very good or good. However, a majority of individuals (*n =* 354, 71.4%) reported having at least one physical health problem confirmed by a doctor. Allergies, gastrointestinal disorders, and the broad group of cardiometabolic conditions were the most common health issues. A total of 195 individuals (39.3%) were currently on medication for their health problem (for example, hypertension, allergies or hypothyroidism). Nearly half of the sample (*n =* 236, 47.6%) reported a BMI ≥ 25 (i.e., overweight or above). Among them, 123 individuals (24.8%) had a BMI ≥ 30 (i.e., obesity).

There were differences by OCD symptom severity in the reporting of gastrointestinal (χ^2^(2) = 9.37, *p* = 0.009) and respiratory conditions (χ^2^(2) = 6.68, *p* = 0.035). Those with severe and moderate OCD presented with more gastrointestinal diseases, compared to individuals with mild OCD. Respiratory diseases were significantly more common in individuals with severe OCD, compared to those with moderate OCD, but the frequencies in these two groups were not different from those with mild OCD. There were gender differences in the reporting of ‘any’ health problems (χ^2^(2) = 10.51 *p* = 0.005), with women reporting a higher prevalence of physical health problems than men. Further, gastrointestinal conditions, migraine headaches, autoimmune diseases, and other health problems were statistically significantly more common in women, compared to men, while cardiometabolic conditions (and within this group, hypertension) were more common in men, compared to women and the ‘other gender’ category. An older age was also associated with an increased likelihood of reporting a health problem (χ^2^(2) = 6.71, *p* = 0.035), particularly cardiometabolic conditions, and a higher BMI (*F*(2,489) = 6.53, *p* = 0.002).

### Lifestyle habits

Table 3 shows the results for the lifestyle habits in the total sample. Results by OCD symptom severity, gender, and age group are shown in **Supplementary Tables 4–6** and summarized below.

**Table 3:** Lifestyle habits of the survey participants (*N* = 496)

Variable	*n* (%)
**Physical activity**	
IPAQ-SF, MET minutes/week, Mean *(SD)*	827.7 (994.3)
Low physical activity	271 (55.0)
Moderate physical activity	143 (29.0)
High physical activity	79 (16.0)
**Sedentary time**	
3 h or less per day	57 (11.5)
4–7 h per day	211 (42.5)
8 h or more per day	194 (39.1)
Don’t know/Not sure	34 (6.9)
**Diet**	
Dietary index score, Mean *(SD)*	5.7 (2.4)
Healthy diet	60 (12.1)
Relatively healthy diet	254 (51.2)
Unhealthy diet	182 (36.7)
Breakfast habits	
Every morning	252 (50.8)
Almost every morning	92 (18.6)
A few times a week	70 (14.1)
Once a week or less	82 (16.5)
Meat consumption	
Twice a day or more often	26 (5.2)
Once a day	80 (16.1)
A few times weekly	225 (45.4)
Once a week or less	165 (33.3)
Salt consumption	
Twice a day or more often	38 (7.7)
Once a day	72 (14.5)
A few times weekly	199 (40.1)
Once a week or less	187 (37.7)
**Tobacco use**	
Tobacco user (cigarettes or snuff/smokeless tobacco)	75 (15.1)
Cigarrette smoker	49 (9.9)
Cigarettes/day, Mean *(SD)*	10.4 (7.9)
Smoked but quit	98 (21.9)
Snuff/smokeless tobacco user	33 (6.7)
Snuff boxes/week, Mean *(SD)*	3.3 (2.9)
Used but quit	17 (3.7)
**Alcohol use**	
AUDIT-C, Mean *(SD)*	1.9 (2.4)
Risk consumption of alcohol	111 (22.3)
**Drug use in the past year**	
Never	414 (83.5)
Less than monthly	41 (8.3)
Monthly	11 (2.2)
Weekly	9 (1.8)
Daily or almost daily	21 (4.2)
**Sleep**	
Average sleep duration per night	
6 h or less	130 (26.2)
7–9 h	308 (62.1)
9 h or more	58 (11.7)
Waking up feeling fresh and rested	
None of the time	124 (25.0)
A little of the time	144 (29.0)
Some of the time	126 (25.4)
A good bit of the time	58 (11.7)
Most of the time	37 (7.5)
All of the time	7 (1.4)

*Abbreviations: AUDIT-C*, Alcohol Use Disorders Identification Test– Concise; *IPAQ-SF*, The International Physical Activity Questionnaire - Short Form; *MET*, Metabolic equivalent of task; *SD*, standard deviation

#### Physical activity

The mean score on the IPAQ-SF was 827.7 MET minutes/week (*SD* = 994.3), which is in the moderate physical activity range. In a categorical classification, 271 individuals (55.0%) were in the low physical activity group, 143 (29.0%) were considered to be moderately active, and 79 (16.0%) were considered to be highly physically active. Similarly, a majority of individuals reported several hours of sedentary time (4–7 h per day, *n* = 211, 42.5%; 8 h or more per day, *n* = 194, 39.1%).

There were statistically significant gender differences in the MET minutes/week (χ^2^(4) = 12.8, *p* = 0.012), with men and ‘other gender’ being more physically active than women. There was also a significant association between age group and physical acitivty (*F(*2,490) = 4.83, *p* = 0.008), with the youngest age group having a significantly higher mean of MET-minutes/week compared to individuals aged 31–45. However, a significantly higher proportion of individuals in the youngest age group reported sitting 8 h or more every day, compared to the two older age groups (χ^2^(6) = 13.12, *p* = 0.041). No significant associations were found between sedentary time and OCD severity and gender.

#### Dietary habits

The mean dietary index score for the whole group fell into to the relatively healthy diet category (mean = 5.7, *SD* = 2.4). However, more than one third of participants *(n =* 182, 36.7%) were classified as having unhealthy food habits. Only sixty (12.1%*)* individuals reported answers corresponding to having healthy food habits and 254 (51.2%*)* were classified as having relatively healthy food habits, according to the Swedish National Board of Health and Welfare definition.

There was a significant association between dietary habits and OCD severity (χ^2^(4) = 9.72, *p* = 0.045), with individuals with severe and moderate OCD having a worse diet that those with mild OCD.

#### Tobacco use

In our sample, 75 individuals (15.1%) were tobacco users (including both cigarettes or snuff/smokeless tobacco). Of these, 49 individuals (9.9%) reported to smoke cigarettes and 33 (6.7%) were currently using snuff or smokeless tobacco. A total of 7 individuals (1.4%) both smoked tobacco and used snuff.

A significant association between tobacco use and OCD symptom severity (χ^2^(2) = 7.1, *p* = 0.029) was found. Individuals with more severe OCD were more likely to smoke or use snuff, compared to individuals with mild and moderate OCD.

#### Alcohol and drug use

Concerning alcohol use, 180 individuals (36.3%) reported never drinking alcohol. The mean of AUDIT-C score for the whole sample was 1.9 *(SD* = 2.4), corresponding to a low-risk consumption. Most participants (*n =* 198, 62.7%) usually had 1 to 2 drinks per occasion, and never or less than monthly had 6 or more drinks at the same occasion (*n =* 274, 86.7%). About one fifth of the sample (*n =* 111, 22.3%) were considered to be at risk consumption of alcohol. A majority (*n =* 414, 83.5%) reported that they had not used illegal drugs during the past year. Thirty individuals (6.0%) reported using illicit drugs weekly or daily.

Subgroup analyses showed that the amount of alcohol per occasion was associated with the age group (χ^2^(8) = 26.09, *p* = 0.001), with binge drinking (6 or more drinks per occasion) being less common in the two older age groups. There was also a significant association with drug use and age (χ^2^(8) = 33.72, *p* < 0.001), with drug use being more common in the youngest age group compared to the two older groups. No significant associations were found between alcohol or drug use and OCD severity and gender.

#### Sleep habits

Most participants (*n =* 308, 62.1%) reported sleeping an average of 7–9 h/night. When asked about how often they woke up feeling rested, more than half of the participants (*n =* 268, 54%) responded that it occurred none of the time or a little of the time.

There was a significant association between OCD symptom severity and both sleep time (χ^2^(4) = 25.38, *p* < 0.001) and sleep quality (χ^2^(10) = 19.58, *p* = 0.033), by which more severe OCD was associated with fewer hours of sleep and less restorative sleep, compared to the moderate and mild groups. No significant associations were found between sleep habits and gender and age.

### Attitudes towards lifestyle changes

Attitudes towards and experiences of lifestyle changes in the total sample are shown in Table 4. A large majority of the sample (*n =* 444, 89.5%) reported that they had thought about changing their lifestyle habits. They mainly reported a desire of increasing their physical activity (*n =* 383, 77.2%), eat healthier (*n =* 310, 62.5%), and improve their sleep (*n =* 251, 50.6%). Among those who did not consider changing their lifestyle habits (*n =* 52, 10.5%), the main reasons were that they were hindered by their OCD (*n* = 21, 42.9%) or that they were already satisfied with their current lifestyle (*n* = 10, 20.4%).

**Table 4:** Attitudes towards and experiences of lifestyle changes of the survey participants (*N* = 496)

Variable	*n* (%)
**Considered changing lifestyle habits**	444 (89.5)
If yes: Lifestyle habit that wants to be changed*	
Increase physical activity	383 (77.2)
Eat healthier	310 (62.5)
Sleep better	251 (50.6)
Drink less alcohol or quit drinking alcohol	47 (9.5)
Quit smoking	29 (5.6)
Quit drug consumption	16 (3.2)
Other	35 (7.1)
If no: Main reason for not considering lifestyle change	
Hindered to make lifestyle changes because of OCD	21 (42.9)
Satisfied with current lifestyle	10 (20.4)
Hindered to make lifestyle changes because of other mental health problems	8 (16.3)
No time to incorporate changes to my lifestyle	7 (14.3)
Too expensive to make lifestyle changes	3 (6.1)
**Previously tried to change lifestyle habits**	447 (90.1)
If yes: Succeeded with lifestyle change	246 (55.0)

*Abbreviation: OCD*, obsessive-compulsive disorder

*Participants could select more than one option and therefore the total number does not add up to 100%

Most participants (*n =* 447, 90.1%) had previously tried to change their lifestyle habits, and only around half of these (*n =* 246, 55.0%) had succeeded. From the open text questions, we gathered that increasing physical activity and changes in diet were the most common lifestyle habits that they had previously changed. Participants that had not succeeded in achieving changes mentioned that it was due, for example, to lack of motivation, difficulties in making the changes last or due to their mental health.

## Discussion

The aim of this international survey was to explore the physical health and lifestyle habits of individuals that self-reported a diagnosis of OCD. Our sample consisted of predominantly women, with an age ranging between 18 and 79 years, mainly from Europe and North America, relatively highly educated, and mostly employed or in education. Their self-reported OCD symptom severity was in the moderate range and a vast majority had at least one other psychiatric comorbidity, in line with epidemiological studies [1]. Furthermore, more than 60% were on psychiatric medication and more than 40% were receiving psychological treatment for their OCD.

Despite a self-perception of good health reported by about two thirds of the surveyed participants, more than 70% had been diagnosed with at least one somatic health problem, which replicates findings from previous observational studies indicating that physical health issues are common in individuals with OCD [7]. The most frequent health problems were allergies (about 40% of the sample). The current literature regarding the association between OCD and allergies is scarce, inconsistent, and methodologically limited. A clinical study [56] showed a significantly higher percentage of allergies in an OCD group (61.5%), compared to controls (22.9%). However, the sample sizes in this study were very small (13 vs. 35, respectively). By contrast, in a survey-based study, allergies were not more frequent in individuals with OCD than in controls [17]. The second most reported somatic health conditions in our sample were gastrointestinal disorders, in line with previous preliminary evidence on this association [13, 16]. Cardiometabolic conditions were also commonly reported in our survey. Additionally, nearly half of the participants reported being overweight or obese, a known risk factor for cardiometabolic conditions, cancer, and type 2 diabetes [57]. The evidence for the association between OCD and cardiovascular and metabolic conditions is now robust [14, 15, 58].

While many of the participants in our sample had relatively healthy lifestyle habits, a significant proportion reported low physical activity, unhealthy dietary habits, risk consumption of alcohol, and a non-restorative sleep. Half of the sample (55%) did not meet recommendations of weekly physical activity, which is a higher proportion than the 27% of the global adult population that are classified as not sufficiently active [59]. Our results are similar to those reported by Aguglia et al. [13], who described a lack of physical activity in 36% and only low levels of physical activity in 41% of 162 individuals with OCD. Furthermore, individuals with general medical conditions in that study were more likely to have lower levels of activity [13]. Because this was a cross-sectional study, directionality could not be established, but regular physical activity is known to be an important factor for improving physical health and preventing disease, such as cardiovascular diseases and type 2 diabetes [60, 61]. Additionally, even small increases in physical activity levels can improve cardiorespiratory fitness, that in turn are linked to significantly lower risk of all-cause mortality and cardiovascular events [62]. There is also evidence of mental health benefits, such as reduced anxiety and depressive symptoms, from increased physical activity [63, 64]. To date, only a handful of studies have tested with promising results the effect of physical activity on OCD symptoms, including one small randomized controlled trial of 56 participants [65, 66]. The exercise intervention also increased the participants’ physical activity levels and their cardiorespiratory fitness [65], indicating that interventions aiming to increase physical activity in individuals with OCD are feasible. However, this controlled study was underpowered and therefore the efficacy of this intervention is still unclear.

Over one third of the participants (37%) in our sample were on an unhealthy diet, defined as a low intake of vegetables, fruits and fish, and a high intake of sugary foods. Poor dietary habits have shown to be responsible for more deaths globally than tobacco [67], stressing the need to target and improve a suboptimal diet. The proportion of individuals with an unhealthy diet in our total sample is larger than that with an unhealthy diet in Sweden (20%) [41]. However, in the United States, almost half of the population (46%) are estimated to have a poor-quality diet [68]. Nonetheless, most respondents in our survey seemed to have at least relatively healthy dietary habits, in line with the findings by Nguyen et al. [24] on overall dietary quality in OCD, where, in a sample of 85 individuals with OCD, most were considered to meet dietary recommendations.

In our sample, 22% of individuals with OCD were classified as having a risky alcohol consumption. Population data on prevalence of harmful alcohol consumption varies by country, gender, and age, but was estimated to be 17% in 2020 globally [69]. The WHO reports a prevalence of 18% for heavy episode drinking in a global status report [70], and the average prevalence of risky drinking in the European countries was estimated to 19% in 2019 [71]. Overall, these numbers are not far from those reported by our sample. However, the methodological differences do not allow to make head-to-head comparisons that can establish whether the differences are significant. Nonetheless, previous register-based data using a large cohort of individuals with OCD diagnosed in specialist care in Sweden showed a 3.7-fold increased risk of any substance misuse disorder, including use of alcohol and also other drugs, in individuals with OCD, compared to individuals from the general population [32]. Use of tobacco (cigarettes or snuff/smokeless tobacco) in our sample was around 15%, well in line with current population estimates [26]. Drug use was not common among our surveyed participants, with 84% of participants answering that they had not used drugs in the past year.

Most of our survey participants slept 7 to 9 h in average per night, which would meet international recommendations [72]. However, a majority (54%) reported to have a non-restorative sleep. Sleep disturbances have showed to be common in OCD [18, 19, 33–35]. Reasons for this association are unclear. Some studies suggest that it could be explained by comorbid depression [18, 33, 35] and anxiety disorders [35], while others indicate that depressive symptoms alone do not fully explain the association [19, 34]. In our sample, around half of the individuals reported comorbid depression and anxiety disorders, which could potentially influence sleep quality. While prior research found that individuals with OCD have shorter sleep duration than controls [18, 19], in our survey, most participants reported average sleep durations. However, the lack of a control group and the use of different methods makes it difficult to contextualize this finding. In turn, short duration of sleep and poor sleep quality have been identified as risk factors for the development of obesity and type 2 diabetes [73–75], highlighting the need to target sleep to reduce the risk of medical health problems.

Overall, stratified analyses by OCD symptom severity, gender, and age group showed that groups were similar, with some expected exceptions. More severe OCD symptoms were associated with unhealthier dietary habits, higher tobacco use, fewer hours of sleep, and a non-restorative sleep. This is partly in line with previous research findings showing a significant association between higher obsessive-compulsive symptom severity and lower intake of vegetables and oily fish [76]. These results contrast with those reported by Nguyen et al. [24], who found no association between OCD severity and dietary quality. Nonetheless, head-to-head comparisons are difficult due to methodological differences. Additionally, our results support earlier research highlighting an association between OCD severity and sleep disturbances [33].

Women reported a higher prevalence of physical health problems than men, and there were also differences in the pattern of somatic conditions reported by gender. It was more common for women to report gastrointestinal conditions, migraine headaches, and autoimmune disorders, while men reported more cardiometabolic conditions. Men were more physically active than women, which is also the case in the general population [59]. The oldest group of individuals in this sample reported more somatic health problems and a higher BMI than younger individuals. These results are to be expected since ageing in the general population is a risk factor for different chronic conditions, such as cardiovascular disease, stroke, and cancer [77]. On the other hand, risk consumption of alcohol and drug use was more common in the youngest age group.

Importantly, a large majority of the sample (90%) reported that they had previously tried to change their lifestyle habits, but only around half of them had succeeded. This indicates the importance of supporting this group to make the necessary lifestyle changes, given their proved usefulness in preventing many serious disorders, such as cardiovascular disorders or cancer [78, 79]. Lifestyle interventions have been tested with good results in the general population [80, 81] and in individuals with psychiatric disorders, mainly bipolar disorder, psychotic disorders, and depression [82–84]. To the best of our knowledge, no lifestyle intervention to prevent risk factors associated with medical conditions has been tested in individuals with OCD. Such interventions would be very fitting for this group, given the increased morbidity of health problems and their reported difficulties to incorporate lifestyle changes on their own. It would also contribute to a more holistic model of healthcare for individuals with psychiatric disorders focusing on broader aspects beyond their psychiatric symptoms [5, 23].

The results should be considered in light of several limitations. First, the study used a non-probabilistic sampling method. Furthermore, our sample consisted mostly of women from Europe and North America. The higher proportion of women was expected given that OCD has shown to be more prevalent in women [2], compared to men, and that women are more likely to participate in research studies [85]. However, the results should be replicated in larger samples of men and non-binary/non-conforming individuals, as well as in non-occidental settings. Altogether, these issues limit the generalizability of our results to the whole population of individuals with OCD. Second, the accuracy of self-reported lifestyle habits and health factors can be questioned, as it may be affected by recall and/or response bias. For example, previous studies have shown that self-reported physical activity and height are often overestimated [86–88], whereas weight is underestimated [87, 89]. Third, while most of the questions in the survey evaluating lifestyle habits were taken from validated questionnaires, they were slightly modified to suit the survey structure and to avoid overwhelming the participants with lengthy questions, and translated into the different languages of the survey. Additionally, the questions for tobacco/snuff use and one of the two questions to evaluate sleep were purposely created for the survey and are therefore not validated. Fourth, the accuracy of the OCD diagnosis could not be verified. However, the characteristics of the participants in this study align well with what is often found in epidemiological and clinical samples of individuals with clinically diagnosed OCD, with symptoms (measured with a validated scale) that were in the moderate to severe range for most respondents. Fifth, the study lacked a control group, which makes it difficult to put the findings into context. Finally, a large number of comparisons were made, resulting in a potential risk of making a type I error. However, we did not correct for multiple comparisons due to the exploratory nature of the study and the non-independence of the variables.

In conclusion, this study confirms results from previous literature using different methodologies reporting an association between OCD and somatic health issues. We showed that a significant proportion of participants had unhealthy habits, most prominently a lack of physical activity. Given the association between physical health and lifestyle habits, and the reported difficulties for this group to implement changes, the development and evaluation of interventions aimed to improve the lifestyle habits of individuals with OCD is warranted.

### Electronic supplementary material

Below is the link to the electronic supplementary material.

Supplementary Material 1

## Data Availability

The datasets generated and analyzed during the current study are not publicly available due to decision from the ethical authority, but could be made available from the corresponding author on reasonable request.

## Abbreviations

AUDIT-C: Alcohol Use Disorders Identification Test– Concise
BMI: Body Mass Index
IPAQ-SF: International Physical Activity Questionnaire, short version
OCD: Obsessive-compulsive disorder
OCI-12: 12-item Obsessive-Compulsive Inventory
MET: Metabolic equivalent of task
SD: Standard deviation
SF-36: Short Form of Health Survey
SSRI: Selective serotonin reuptake inhibitors
WHO: World Health Organization
WHO-5: 5-item WHO Well-Being Index
