# Supplementary material for: General somatic health and lifestyle habits in individuals with obsessive- compulsive disorder: an international survey
Source: BMC Psychiatry. 2024 Feb 5;24:98. doi: 10.1186/s12888-024-05566-w (PMC10840209; doi:10.1186/s12888-024-05566-w)
Supplement: Supplementary file 1 — Supplementary Material 1 [file 12888_2024_5566_MOESM1_ESM.docx]

**SUPPLEMENTARY MATERIAL**

**Supplementary Table 1.** Somatic health of the survey participants (N=496), by OCD symptom severity.

| **Variable** | **Severe OCD symptoms**  **(n=228)** | **Moderate**  **OCD symptoms**  **(n=162)** | **Mild OCD symptoms**  **(n=106)** | **χ^2^/*F*** | ***p-*value** |
| --- | --- | --- | --- | --- | --- |
|  | **n (%)** | **n (%)** | **n (%)** |  |  |
| **Self-rated health** |  |  |  | 11.40 | 0.180 |
| Excellent | 8 (3.5) | 6 (3.7) | 7 (6.6) |  |  |
| Very good | 40 (17.5) | 35 (21.6) | 27 (25.5) |  |  |
| Good | 88 (38.6) | 67 (41.4) | 45 (42.5) |  |  |
| Fair | 67 (29.4) | 45 (27.8) | 20 (18.9) |  |  |
| Poor | 25 (10.9) | 9 (5.6) | 7 (6.6) |  |  |
| **Health problems** |  |  |  |  |  |
| Any | 171 (75.0) | 113 (67.8) | 70 (66.0) | 3.15 | 0.207 |
| Allergies | 94 (41.2) | 59 (36.4) | 39 (36.8) | 1.13 | 0.568 |
| Gastrointestinal conditions | 73 (32.0)^a^ | 44(27.2)^a^ | 17(16.0)^b^ | 9.37 | 0.009** |
| Cardiometabolic conditions | 46 (20.2) | 33 (20.4) | 18 (16.9) | 0.57 | 0.752 |
| Hypertension | 26 (11.4) | 18 (11.1) | 14 (13.2) | 0.31 | 0.858 |
| High cholesterol | 27 (11.8) | 18 (11.1) | 7 (6.6) | 2.22 | 0.330 |
| Type 2 diabetes | 8 (3.5) | 7 (4.3) | 3 (2.8) | 0.42 | 0.809 |
| Cardiovascular disorder | 6 (2.6) | 3 (1.9) | 2 (1.9) | 0.33 | 0.846 |
| Previous acute cardiovascular or cerebrovascular event | 0 (0.0) | 1 (0.6) | 1 (0.9) | 0.39 | 0.391 |
| Migraine headaches | 41 (17.9) | 32 (19.8) | 10 (9.4) | 5.37 | 0.068 |
| Musculoskeletal conditions | 37 (16.2) | 28 (17.3) | 9 (8.5) | 4.47 | 0.107 |
| Respiratory conditions | 29 (12.7)^a^ | 8 (4.9)^b^ | 10 (9.4)^ab^ | 6.68 | 0.035* |
| Autoimmune diseases | 12 (11.3) | 14 (8.6) | 18 (7.9) | 1.07 | 0.587 |
| Neurological diseases | 8 (3.5) | 1 (0.6) | 2 (1.9) | 3.72 | 0.156 |
| Cancer | 2 (0.9) | 5 (3.1) | 2 (1.9) | 2.60 | 0.273 |
| Other health problem | 41 (17.9) | 25 (15.4) | 23 (21.7) | 1.71 | 0.425 |
| **Body Mass Index** |  |  |  |  |  |
| *Mean (SD)* | 26.3 (6.1) | 25.9 (5.5) | 26.4 (7.4) | 0.29 | 0.752 |
|  |  |  |  | 13.25 | 0.039* |
| Obese | 31 (29.3)^a^ | 32 (20.0)^a^ | 31 (29.3)^a^ |  |  |
| Overweight | 42 (18.6)^a^ | 48 (30.0)^a^ | 23 (21.7)^a^ |  |  |
| Normal weight | 107 (47.4)^a^ | 76 (47.5)^a^ | 44 (41.5)^a^ |  |  |
| Underweight | 17 (7.5)^a^ | 4 (2.5)^a^ | 8 (7.6)^a^ |  |  |
| **Currently taking medication for physical health issues** | 95 (41.7) | 57 (35.2) | 43 (40.6) | 1.76 | 0.416 |

*Abbreviations:* *OCD,* obsessive-compulsive disorder; *SD*, standard deviation.

*Note:* Frequencies or means with different superscripts differ significantly in paired Chi-squared tests of independence (for categorical variables) or after Tukey or Tamhane’s T2 post-hoc correction (for continuous variables); proportions with differing superscripts differ significantly at p<0.05.

*Significant at p<0.05; **significant at p<0.01.

**Supplementary Table 2.** Somatic health of the survey participants (N=496), by gender.

| **Variable** | **Women (n=391)** | **Men**  **(n=92)** | **Other / prefer not to say**  **(n=13)** | **χ^2^/*F*** | ***p-*value** |
| --- | --- | --- | --- | --- | --- |
|  | **n (%)** | **n (%)** | **n (%)** |  |  |
| **Self-rated health** |  |  |  | 11.05 | 0.199 |
| Excellent | 15 (3.8) | 6 (6.5) | 0 (0.0) |  |  |
| Very good | 83 (21.2) | 15 (16.3) | 4 (30.8) |  |  |
| Good | 149 (38.1) | 44 (47.8) | 7 (53.9) |  |  |
| Fair | 107 (27.4) | 24 (26.1) | 1 (7.7) |  |  |
| Poor | 37 (9.5) | 3 (3.3) | 1 (7.7) |  |  |
| **Health problems** |  |  |  |  |  |
| Any | 291 (74.4)^a^ | 53 (57.6)^b^ | 10 (76.9)^ab^ | 10.51 | 0.005** |
| Allergies | 158 (40.4) | 28 (30.4) | 6 (46.2) | 3.44 | 0.180 |
| Gastrointestinal conditions | 118 (30.2)^a^ | 14 (15.2)^b^ | 2 (15.4)^ab^ | 9.37 | 0.009** |
| Cardiometabolic conditions | 71 (18.2)^a^ | 26 (28.3)^b^ | 0 (0.0)^a^ | 8.08 | 0.018* |
| Hypertension | 36 (9.2)^a^ | 22 (23.9)^b^ | 0 (0.0)^a^ | 17.37 | <0.001** |
| High cholesterol | 39 (9.9) | 13 (14.1) | 0 (0.0) | 2.93 | 0.231 |
| Type 2 diabetes | 14 (3.6) | 4 (4.4) | 0 (0.0) | 0.63 | 0.730 |
| Cardiovascular disorder | 10 (2.6) | 1 (1.1) | 0 (0.0) | 1.05 | 0.593 |
| Previous acute cardiovascular or cerebrovascular event | 2 (0.5) | 0 (0.0) | 0 (0.0) | 0.54 | 0.764 |
| Migraine headaches | 74 (18.9)^a^ | 7 (7.6)^b^ | 2 (15.4)^ab^ | 6.86 | 0.032* |
| Musculoskeletal conditions | 63 (16.1) | 8 (8.7) | 3 (23.1) | 3.93 | 0.140 |
| Respiratory conditions | 42 (10.7) | 4 (4.4) | 1 (7.7) | 3.60 | 0.165 |
| Autoimmune diseases | 41(10.5)^a^ | 1 (1.1)^b^ | 2 (15.4)^a^ | 8.84 | 0.012* |
| Neurological diseases | 10 (2.6) | 0 (0.0) | 1 (7.7) | 4.09 | 0.129 |
| Cancer | 8 (2.1) | 1 (1.1) | 0 (0.0) | 0.63 | 0.729 |
| Other health problem | 79 (20.2)^a^ | 9 (9.8)^b^ | 1(7.7)^ab^ | 6.45 | 0.040* |
| **Body Mass Index** |  |  |  |  |  |
| *Mean (SD)* | 26.0 (6.7) | 26.8 (5.7) | 26.6 (6.6) | 0.57 | 0.569 |
|  |  |  |  | 8.46 | 0.206 |
| Obese | 95 (24.4) | 24 (26.4) | 4 (33.3) |  |  |
| Overweight | 83 (21.3) | 28 (30.8) | 2 (16.7) |  |  |
| Normal weight | 184 (47.3) | 38 (41.8) | 5 (41.7) |  |  |
| Underweight | 27 (6.9) | 1 (1.1) | 1 (8.3) |  |  |
| **Currently taking medication for physical health issues** | 160 (40.9) | 32 (34.8) | 3 (23.1) | 2.65 | 0.266 |

*Abbreviations:* *OCD,* obsessive-compulsive disorder; *SD*, standard deviation.

*Note:* Frequencies or means with different superscripts differ significantly in paired Chi-squared tests of independence (for categorical variables) or after Tukey or Tamhane’s T2 post-hoc correction (for continuous variables); proportions with differing superscripts differ significantly at p<0.05.

*Significant at p<0.05; **significant at p<0.01.

**Supplementary Table 3.** Somatic health of the survey participants (N=496), by age group.

| **Variable** | **18-30 years**  **(n=196)** | **31-45 years**  **(n=180)** | **≥46 years**  **(n=120)** | **χ^2^/*F*** | ***p-*value** |
| --- | --- | --- | --- | --- | --- |
|  | **n (%)** | **n (%)** | **n (%)** |  |  |
| **Self-rated health** |  |  |  | 9.11 | 0.333 |
| Excellent | 8 (4.1) | 5 (2.8) | 8 (6.7) |  |  |
| Very good | 50 (25.5) | 34 (18.9) | 18 (15.0) |  |  |
| Good | 77 (39.3) | 76 (42.2) | 47 (39.2) |  |  |
| Fair | 45 (22.9) | 50 (27.8) | 37 (30.8) |  |  |
| Poor | 16 /8.2) | 15 (8.3) | 10 (8.3) |  |  |
| **Health problems** |  |  |  |  |  |
| Any | 128 (65.3)^a^ | 132 (73.3)^ab^ | 94 (78.3)^b^ | 6.71 | 0.035* |
| Allergies | 70 (35.7) | 75 (41.7) | 47 (39.2) | 1.42 | 0.493 |
| Gastrointestinal conditions | 46 (23.5) | 47 (26.1) | 41 (34.2) | 4.44 | 0.109 |
| Cardiometabolic conditions | 12 (6.1)^a^ | 32 (17.8)^b^ | 53 (44.2)^c^ | 69.04 | <0.001** |
| Hypertension | 6 (3.1)^a^ | 13 (7.2)^a^ | 39 (32.5)^b^ | 67.94 | <0.001** |
| High cholesterol | 6 (3.1)^a^ | 17 (9.4)^b^ | 29 (24.2)^c^ | 35.65 | <0.001** |
| Type 2 diabetes | 0 (0.0)^a^ | 5 (2.8)^b^ | 13 (10.8)^c^ | 25.56 | <0.001** |
| Cardiovascular disorder | 3 (1.5) | 4 (2.2) | 4(3.3) | 1.12 | 0.573 |
| Previous acute cardiovascular or cerebrovascular event | 0 (0.0) | 0 (0.0) | 2 (1.7) | 6.29 | 0.043* |
| Migraine headaches | 27 (13.8) | 35 (19.4) | 21 (17.5) | 2.23 | 0.328 |
| Musculoskeletal conditions | 18 (9.2)^a^ | 28 (15.6)^ab^ | 28 (23.3)^b^ | 11.83 | 0.003** |
| Respiratory conditions | 19 (9.7) | 18 (10.0) | 10 (8.3) | 0.25 | 0.882 |
| Autoimmune diseases | 13 (6.6)^a^ | 13 (7.2)^a^ | 18 (15.0)^b^ | 7.40 | 0.025* |
| Neurological diseases | 7 (3.6) | 2 (1.1) | 2 (1.7) | 2.84 | 0.242 |
| Cancer | 1 (0.5) | 4 (2.2) | 4 (3.3) | 3.59 | 0.166 |
| Other health problem | 31 (15.8) | 37 (20.6) | 21 (17.5) | 1.45 | 0.484 |
| **Body Mass Index** |  |  |  |  |  |
| *Mean (SD)* | 24.9 (6.4)^a^ | 26.8 (6.5)^b^ | 27.3 (6.6)^b^ | 6.53 | 0.002** |
|  |  |  |  | 13.05 | 0.042* |
| Obese | 39 (20.0)^a^ | 50 (27.9)^a^ | 34 (28.8)^a^ |  |  |
| Overweight | 39 (20.0)^a^ | 42 (23.5)^a^ | 32 (27.1)^a^ |  |  |
| Normal weight | 99 (50.8)^a^ | 81 (45.3)^a^ | 47 (39.8)^a^ |  |  |
| Underweight | 18 (9.2)^a^ | 6 (3.4)^b^ | 5 (4.2)^ab^ |  |  |
| **Currently taking medication for physical health issues** | 51 (26.0)^a^ | 66 (36.7)^a^ | 78 (65.0)^a^ | 48.23 | <0.001** |

*Abbreviations:* *OCD,* obsessive-compulsive disorder; *SD*, standard deviation.

*Note:* Frequencies or means with different superscripts differ significantly in paired Chi-squared tests of independence (for categorical variables) or after Tukey or Tamhane’s T2 post-hoc correction (for continuous variables); proportions with differing superscripts differ significantly at p<0.05.

*Significant at p<0.05; **significant at p<0.01.

**Supplementary Table 4.** Lifestyle habits of the survey participants (N=496), by OCD symptom severity.

| **Variable** | **Severe OCD symptoms**  **(n=228)** | **Moderate**  **OCD symptoms**  **(n=162)** | **Mild OCD symptoms**  **(n=106)** | **χ^2^/*F*** | ***p-*value** |
| --- | --- | --- | --- | --- | --- |
|  | **n (%)** | **n (%)** | **n (%)** |  |  |
| **Physical activity** |  |  |  |  |  |
| IPAQ-SF MET-minutes/week, Mean *(SD)* | 937.9 (1157.8) | 759.2 (810.7) | 697.0 (841.1) | 2.70 | 0.068 |
|  |  |  |  | 2.94 | 0.568 |
| Low physical activity | 117 (51.8) | 90 (55.9) | 64 (60.4) |  |  |
| Moderate physical activity | 70 (30.9) | 48 (29.8) | 25 (23.6) |  |  |
| High physical activity | 39 (17.3) | 23 (14.3) | 17 (16.0) |  |  |
| **Sedentary time** |  |  |  | 2.83 | 0.829 |
| 3 hours or less per day | 23 (10.1) | 21 (13.0) | 13 (12.3) |  |  |
| 4-7 hours per day | 97 (42.5) | 68 (42.0) | 46 (43.4) |  |  |
| 8 hours or more per day | 90 (39.5) | 61 (37.7) | 43 (40.6) |  |  |
| Don’t know/Not sure | 18 (7.9) | 12 (7.4) | 4 (3.8) |  |  |
| **Diet** |  |  |  |  |  |
| Dietary index score, Mean *(SD)* | 5.6 (2.4) | 5.8 (2.3) | 5.9 (2.4) | 2.23 | 0.108 |
|  |  |  |  | 9.72 | 0.045* |
| Healthy diet | 27 (11.8)^a^ | 22 (13.6)^a^ | 11 (10.4)^a^ |  |  |
| Relatively healthy diet | 111 (48.7)^a^ | 75 (46.3)^a^ | 68 (64.2)^b^ |  |  |
| Unhealthy diet | 90 (39.5)^a^ | 65 (40.1)^a^ | 27(25.5)^b^ |  |  |
| Breakfast habits |  |  |  | 20.34 | 0.002** |
| Every morning | 106 (46.5)^a^ | 85 (52.5)^a^ | 61 (57.6)^a^ |  |  |
| Almost every morning | 39 (17.1)^a^ | 32 (19.8)^a^ | 21 (19.8)^a^ |  |  |
| A few times a week | 28 (12.3)^a^ | 24 (14.8)^a^ | 18 (16.0)^a^ |  |  |
| Once a week or less | 55 (24.1)^a^ | 21 (13.0)^b^ | 6 (5.7)^b^ |  |  |
| Meat consumption |  |  |  | 4.71 | 0.582 |
| Twice a day or more often | 13 (5.7) | 6 (3.7) | 7 (6.6) |  |  |
| Once a day | 40 (17.5) | 25 (15.4) | 15 (14.2) |  |  |
| A few times weekly | 93 (40.8) | 81 (50.0) | 51 (48.1) |  |  |
| Once a week or less | 82 (36.0) | 50 (30.9) | 33 (31.1) |  |  |
| Salt consumption |  |  |  | 4.46 | 0.615 |
| Twice a day or more often | 16 (7.0) | 16 (9.9) | 8 (5.7) |  |  |
| Once a day | 36 (15.8) | 19 (11.7) | 17 (16.0) |  |  |
| A few times weekly | 94 (41.2) | 67 (41.4) | 38 (35.9) |  |  |
| Once a week or less | 82 (36.0) | 60 (37.0) | 45 (42.5) |  |  |
| **Tobacco use** |  |  |  |  |  |
| Tobacco user (cigarettes or snuff/smokeless tobacco) | 45(19.7)^a^ | 19 (11.7)^b^ | 11 (10.4)^b^ | 7.10 | 0.029* |
| Cigarrette smoker | 30 (13.2) | 12 (7.4) | 7 (6.6) | 5.14 | 0.076 |
| Cigarettes/day, Mean *(SD)* | 11.1 (8.5) | 8.8 (6.9) | 9.9 (7.9) | 0.39 | 0.678 |
| Smoked but quit | 54 (27.3)^a^ | 32 (21.3)^ab^ | 12 (12.1)^b^ | 8.90 | 0.012* |
| Snuff/smokeless tobacco user | 20 (8.8) | 8 (4.9) | 5 (4.7) | 3.06 | 0.217 |
| Snuff boxes/week, Mean *(SD)* | 3.6 | 2.8 | 3 | 0.22 | 0.806 |
| Used but quit | 8 (3.9) | 6 (3.9) | 3 (2.9) | 0.18 | 0.914 |
| **Alcohol use** |  |  |  |  |  |
| AUDIT-C, *mean (SD)* | 2.0 (2.5) | 1.9 (2.1) | 2.1 (2.2) | 0.25 | 0.782 |
| Risk consumption of alcohol | 55 (24.1) | 32 (19.8) | 24 (22.6) | 1.05 | 0.593 |
| **Drug use in the past year** |  |  |  | 5.59 | 0.693 |
| Never | 183 (80.3) | 138 (85.2) | 93 (87.7) |  |  |
| Less than monthly | 21 (9.2) | 12 (7.4) | 8 (7.6) |  |  |
| Monthly | 5 (2.2) | 4 (2.5) | 2 (1.9) |  |  |
| Weekly | 5 (2.2) | 3 (1.9) | 1 (0.9) |  |  |
| Daily or almost daily | 14 (6.1) | 5 (3.1) | 2 (1.9) |  |  |
| **Sleep** |  |  |  |  |  |
| Average sleep duration per night |  |  |  | 25.38 | <0.001** |
| 6 hours or less | 81 (35.5)^a^ | 31 (19.1)^b^ | 18 (16.9)^b^ |  |  |
| 7-9 hours | 115 (50.4)^a^ | 116 (71.6)^b^ | 77 (72.6)^b^ |  |  |
| 9 hours or more | 32 (14.0)^a^ | 15 (9.3)^a^ | 11 (10.4)^a^ |  |  |
| Waking up feeling fresh and rested |  |  |  | 19.58 | 0.033* |
| None of the time | 74 (32.5)^a^ | 30 (18.5)^b^ | 20 (18.9)^b^ |  |  |
| A little of the time | 66 (28.9)^a^ | 51 (31.5)^a^ | 27 (25.8)^a^ |  |  |
| Some of the time | 50 (21.9)^a^ | 42 (25.9)^a^ | 34 (32.1)^a^ |  |  |
| A good bit of the time | 20 (8.8)^a^ | 22 (13.6)^a^ | 16 (15.1)^a^ |  |  |
| Most of the time | 16 (7.0)^a^ | 15 (9.3)^a^ | 6 (5.7)^a^ |  |  |
| All of the time | 2 (0.9)^a^ | 2 (1.2)^a^ | 3 (2.8)^a^ |  |  |

*Abbreviations: AUDIT-C,* The Alcohol Use Disorders Identification Test;*IPAQ-SF,* The International Physical Activity Questionnaire - Short Form*; MET,* Metabolic equivalent of task

*Note:* Frequencies or means with different superscripts differ significantly in paired Chi-squared tests of independence (for categorical variables) or after Tukey or Tamhane’s T2 post-hoc correction (for continuous variables); proportions with differing superscripts differ significantly at p<0.05.

*Significant at p<0.05; **significant at p<0.01.

**Supplementary Table 5.** Lifestyle habits of the survey participants (N=496), by gender.

| **Variable** | **Women (n=391)** | **Men**  **(n=92)** | **Other / prefer not to say**  **(n=13)** | **χ^2^/*F*** | ***p-*value** |
| --- | --- | --- | --- | --- | --- |
|  | **n (%)** | **n (%)** | **n (%)** |  |  |
| **Physical activity** |  |  |  |  |  |
| IPAQ-SF MET-minutes/week, Mean *(SD)* | 780.6 (970..3) | 1030.8 (1093.6) | 798.6 (843.0) | 2.37 | 0.094 |
|  |  |  |  | 12.80 | 0.012* |
| Low physical activity | 223 (57.5)^a^ | 41 (44.6)^b^ | 7 (53.9)^ab^ |  |  |
| Moderate physical activity | 112 (28.9)^a^ | 30 (32.6)^a^ | 1 (7.7)^a^ |  |  |
| High physical activity | 53 (13.7)^a^ | 21 (22.8)^b^ | 5 (38.5)^b^ |  |  |
| **Sedentary time** |  |  |  | 1.25 | 0.975 |
| 3 hours or less per day | 47 (12.0) | 9 (9.8) | 1 (7.7) |  |  |
| 4-7 hours per day | 168 (43.0) | 37 (40.2) | 6 (46.2) |  |  |
| 8 hours or more per day | 149 (38.1) | 40 (43.5) | 5 (38.5) |  |  |
| Don’t know/Not sure | 27 (6.9) | 6 (6.5) | 1 (7.7) |  |  |
| **Diet** |  |  |  |  |  |
| Dietary index score, Mean *(SD)* | 5.5 (2.5) | 5.4 (2.5) | 4.8 (1.8) | 0.71 | 0.491 |
|  |  |  |  | 2.27 | 0.686 |
| Healthy diet | 50 (12.8) | 10 (10.9) | 0 (0.0) |  |  |
| Relatively healthy diet | 197 (50.4) | 49 (53.3) | 8 (61.5) |  |  |
| Unhealthy diet | 144 (36.8) | 33 (35.9) | 5 (38.5) |  |  |
| Breakfast habits |  |  |  | 4.13 | 0.659 |
| Every morning | 200 (51.2) | 45 (48.9) | 7 (53.9) |  |  |
| Almost every morning | 72 (18.4) | 18 (19.6) | 2 (15.4) |  |  |
| A few times a week | 50 (12.8) | 18 (19.6) | 2 (15.4) |  |  |
| Once a week or less | 69 (17.7) | 11 (11.9) | 7 (53.9) |  |  |
| Meat consumption |  |  |  | 19.38 | 0.004** |
| Twice a day or more often | 19 (4.9)^a^ | 7 (7.6)^a^ | 0 (0.0)^a^ |  |  |
| Once a day | 51 (13.0)^a^ | 26 (28.3)^b^ | 3 (23.1)^ab^ |  |  |
| A few times weekly | 178 (45.5)^a^ | 41 (44.6)^a^ | 6 (46.2)^a^ |  |  |
| Once a week or less | 143 (36.6)^a^ | 18 (19.6)^b^ | 4 (30.8)^ab^ |  |  |
| Salt consumption |  |  |  | 13.93 | 0.030* |
| Twice a day or more often | 30 (7.7)^a^ | 5 (5.4)^a^ | 3 (23.1)^b^ |  |  |
| Once a day | 52 (13.3)^a^ | 18 (19.6)^a^ | 2 (15.4)^a^ |  |  |
| A few times weekly | 149 (38.1)^a^ | 45 (48.9)^a^ | 5 (38.5)^a^ |  |  |
| Once a week or less | 160 (40.9)^a^ | 24 (26.1)^b^ | 3 (23.1)^ab^ |  |  |
| **Tobacco use** |  |  |  |  |  |
| Tobacco user (cigarettes or snuff/smokeless tobacco) | 56 (14.3) | 18 (19.6) | 1 (7.7) | 2.17 | 0.338 |
| Cigarrette smoker | 37 (9.5) | 11 (11.9) | 1 (7.7) | 0.59 | 0.744 |
| Cigarettes/day, Mean *(SD)* | 9.2 (6.6) | 13.5 (11.0) | *-* | 2.13 | 0.130 |
| Smoked but quit | 80 (20.5) | 17 (18.5) | 1 (7.7) | 2.05 | 0.726 |
| Snuff/smokeless tobacco user | 25 (6.4) | 8 (8.7) | 0 (0.0) | 1.59 | 0.452 |
| Snuff boxes/week, Mean *(SD)* | 3.7 (3.3) | 2 (1.4) | - | 1.97 | 0.171 |
| Used but quit | 13 (3.3) | 4 (4.4) | 0 (0.0) | 2.41 | 0.661 |
| **Alcohol use** |  |  |  |  |  |
| AUDIT-C, *mean (SD)* | 1.9 (2.3) | 2.3 (2.8) | 1.5 (1.7) | 1.13 | 0.325 |
| Risk consumption of alcohol | 85 (21.7) | 23 (25.0) | 3 (23.1) | 0.46 | 0.795 |
| **Drug use in the past year** |  |  |  | 12.59 | 0.127 |
| Never | 328 (83.9) | 76 (82.6) | 10 (76.9) |  |  |
| Less than monthly | 32 (8.2) | 6 (6.5) | 3 (23.1) |  |  |
| Monthly | 6 (1.5) | 5 (5.4) | 0 (0.0) |  |  |
| Weekly | 6 (1.5) | 3 (3.3) | 0 (0.0) |  |  |
| Daily or almost daily | 19 (4.9) | 2 (2.2) | 0 (0.0) |  |  |
| **Sleep** |  |  |  |  |  |
| Average sleep duration per night |  |  |  | 4.64 | 0.326 |
| 6 hours or less | 102 (26.1) | 22 (23.9) | 6 (46.2) |  |  |
| 7-9 hours | 240 (61.4) | 61 (66.3) | 7 (53.9) |  |  |
| 9 hours or more | 49 (12.5) | 9 (9.8) | 0 (0.0) |  |  |
| Waking up feeling fresh and rested |  |  |  | 10.17 | 0.426 |
| None of the time | 98 (25.1) | 22 (23.9) | 4 (30.8) |  |  |
| A little of the time | 121 (30.9) | 19 (20.7) | 4 (30.8) |  |  |
| Some of the time | 93 (23.8) | 31 (33.7) | 2 (15.4) |  |  |
| A good bit of the time | 44 (11.3) | 12 (13.0) | 2 (15.4) |  |  |
| Most of the time | 31 (7.9) | 5 (5.4) | 1 (7.7) |  |  |
| All of the time | 4 (1.0) | 3 (3.3) | 0 (0.0) |  |  |

*Abbreviations: AUDIT-C,* The Alcohol Use Disorders Identification Test;*IPAQ-SF,* The International Physical Activity Questionnaire - Short Form*; MET,* Metabolic equivalent of task

*Note:* Frequencies or means with different superscripts differ significantly in paired Chi-squared tests of independence (for categorical variables) or after Tukey or Tamhane’s T2 post-hoc correction (for continuous variables); proportions with differing superscripts differ significantly at p<0.05.

*Significant at p<0.05; **significant at p<0.01.

**Supplementary Table 6.** Lifestyle habits of the survey participants (N=496), by age group.

| **Variable** | **18-30 years**  **(n=196)** | **31-45 years**  **(n=180)** | **≥46 years**  **(n=120)** | **χ^2^/*F*** | ***p-*value** |
| --- | --- | --- | --- | --- | --- |
|  | **n (%)** | **n (%)** | **n (%)** |  |  |
| **Physical activity** |  |  |  |  |  |
| IPAQ-SF MET-minutes/week, Mean *(SD)* | 990.3 (1149.5)^a^ | 677.4 (771.9)^b^ | 787.3 (985.3)^ab^ | 4.83 | 0.008** |
|  |  |  |  | 7.91 | 0.095 |
| Low physical activity | 100 (51.3) | 103 (57.5) | 68 (57.1) |  |  |
| Moderate physical activity | 53 (27.2) | 56 (31.3) | 34 (28.6) |  |  |
| High physical activity | 42 (21.5) | 20 (11.2) | 17 (14.3) |  |  |
| **Sedentary time** |  |  |  | 13.12 | 0.041* |
| 3 hours or less per day | 16 (8.2)^a^ | 24 (13.3)^a^ | 17 (14.2)^a^ |  |  |
| 4-7 hours per day | 78 (39.8)^a^ | 80 (44.4)^a^ | 53 (44.2)^a^ |  |  |
| 8 hours or more per day | 92 (46.9)^a^ | 65 (36.1)^b^ | 37 (30.8)^b^ |  |  |
| Don’t know/Not sure | 10 (5.1)^a^ | 11 (6.1)^a^ | 13 (10.8)^a^ |  |  |
| **Diet** |  |  |  |  |  |
| Dietary index score, Mean *(SD)* | 5.4 (2.5) | 5.4 (2.4) | 5.8 (2.7) | 1.33 | 0.265 |
|  |  |  |  | 2.75 | 0.600 |
| Healthy diet | 20 (10.2) | 21 (11.7) | 19 (15.8) |  |  |
| Relatively healthy diet | 101 (51.5) | 96 (53.3) | 57 (47.5) |  |  |
| Unhealthy diet | 75 (38.3) | 63 (35.0) | 44 (36.7) |  |  |
| Breakfast habits |  |  |  | 14.79 | 0.022* |
| Every morning | 86 (43.9)^a^ | 92 (51.1)^ab^ | 74 (61.7)^b^ |  |  |
| Almost every morning | 46 (23.5)^a^ | 26 (14.4)^b^ | 20 (16.7)^ab^ |  |  |
| A few times a week | 32 (16.3)^a^ | 25 (13.9)^a^ | 13 (10.8)^a^ |  |  |
| Once a week or less | 32 (16.3)^ab^ | 37 (20.6)^a^ | 13 (10.8)^b^ |  |  |
| Meat consumption |  |  |  | 20.00 | 0.003** |
| Twice a day or more often | 15 (7.7)^a^ | 9 (5.0)^ab^ | 2 (1.7)^b^ |  |  |
| Once a day | 33 (16.8)^ab^ | 36 (20.0)^a^ | 11 (9.2)^b^ |  |  |
| A few times weekly | 74 (37.8)^a^ | 80 (44.4)^a^ | 71 (59.2)^b^ |  |  |
| Once a week or less | 74 (37.8)^a^ | 55 (30.6)^a^ | 36 (30.0)^a^ |  |  |
| Salt consumption |  |  |  | 6.13 | 0.409 |
| Twice a day or more often | 19 (9.7) | 14 (7.8) | 5 (4.2) |  |  |
| Once a day | 32 (16.3) | 26 (14.4) | 14 (11.7) |  |  |
| A few times weekly | 70 (35.7) | 76 (42.2) | 53 (44.2) |  |  |
| Once a week or less | 75 (38.3) | 64 (35.6) | 48 (40.0) |  |  |
| **Tobacco use** |  |  |  |  |  |
| Tobacco user (cigarettes or snuff/smokeless tobacco) | 23 (11.7) | 33 (18.3) | 19 (15.8) | 3.25 | 0.197 |
| Cigarrette smoker | 14 (7.1) | 18 (10.0) | 17 (14.2) | 4.13 | 0.127 |
| Cigarettes/day, Mean *(SD)* | 6.8 (6.4) | 13.1 (7.7) | 10.5 (8.6) | 2.61 | 0.084 |
| Smoked but quit | 33 (16.8) | 40 (22.2) | 25 (20.8) | 6.74 | 0.150 |
| Snuff/smokeless tobacco user | 12 (6.1) | 16 (8.9) | 5 (4.2) | 2.73 | 0.255 |
| Snuff boxes/week, Mean *(SD)* | 2.8 (2.1) | 4 (3.5) | 2 (2.8) | 1.06 | 0.359 |
| Used but quit | 6 (3.1) | 6 (3.3) | 5 (4.2) | 2.97 | 0.562 |
| **Alcohol use** |  |  |  |  |  |
| AUDIT-C, *mean (SD)* | 2.2 (2.4) | 1.9 (2.4) | 1.7 (2.0) | 2.02 | 0.134 |
| Risk consumption of alcohol | 57 (29.1)^a^ | 33(18.3)^b^ | 21 (17.5)^b^ | 8.41 | 0.015* |
| **Drug use in the past year** |  |  |  | 33.72 | <0.001** |
| Never | 142 (72.5)^a^ | 160 (88.9)^b^ | 112 (93.3)^b^ |  |  |
| Less than monthly | 27 (13.8)^a^ | 8 (4.4)^b^ | 6 (5.0)^b^ |  |  |
| Monthly | 8 (4.1)^a^ | 3 (1.7)^ab^ | 0 (0.0)^b^ |  |  |
| Weekly | 4 (2.0)^a^ | 3 (1.7)^a^ | 2 (1.7)^a^ |  |  |
| Daily or almost daily | 15 (7.7)^a^ | 6 (3.3)^a^ | 0 (0.0)^b^ |  |  |
| **Sleep** |  |  |  |  |  |
| Average sleep duration per night |  |  |  | 7.04 | 0.134 |
| 6 hours or less | 41 (20.9) | 48 (26.7) | 41 (34.2) |  |  |
| 7-9 hours | 131 (66.8) | 112 (62.2) | 65 (54.2) |  |  |
| 9 hours or more | 24 (12.2) | 20 (11.1) | 14 (11.7) |  |  |
| Waking up feeling fresh and rested |  |  |  | 9.84 | 0.455 |
| None of the time | 47 (23.9) | 47 (26.1) | 30 (25.0) |  |  |
| A little of the time | 64 (32.7) | 53 (29.4) | 27 (22.5) |  |  |
| Some of the time | 44 (22.5) | 51 (28.3) | 31 (25.8) |  |  |
| A good bit of the time | 23 (11.7) | 19 (10.6) | 16 (13.3) |  |  |
| Most of the time | 15 (7.7) | 8 (4.4) | 14 (11.7) |  |  |
| All of the time | 3 (1.5) | 2 (1.1) | 2 (1.7) |  |  |

*Abbreviations: AUDIT-C,* The Alcohol Use Disorders Identification Test;*IPAQ-SF,* The International Physical Activity Questionnaire - Short Form*; MET,* Metabolic equivalent of task

*Note:* Frequencies or means with different superscripts differ significantly in paired Chi-squared tests of independence (for categorical variables) or after Tukey or Tamhane’s T2 post-hoc correction (for continuous variables); proportions with differing superscripts differ significantly at p<0.05.

*Significant at p<0.05; **significant at p<0.01.

# **Supplementary material- Survey**

# Lifestyle habits and physical health in OCD

In this global survey, we are interested in exploring lifestyle habits and physical health in adults with a diagnosis of obsessive-compulsive disorder (OCD) from all over the world.

What are your lifestyle habits? Do you have any physical health problems? Have you ever tried to change your lifestyle habits? Or are you satisfied with your current lifestyle? 

The results of the survey can lead to a better understanding of the physical health and lifestyle habits (physical activity, diet, alcohol and tobacco use) in people with OCD.

You are welcome to participate if you are at least 18 years old and if you agree that we use your anonymous answers for research purposes. Your answers are completely anonymous and no identifiable information will be stored anywhere. Your participation is completely voluntary. The survey will take approximately 15 minutes to complete.

This study has been approved by the Swedish Ethical Review Authority (register number 2021-02608).

We thank you in advance for your participation. 

Sincerely, 
Dr Lorena Fernández de la Cruz, PhD
Principal Investigator
Karolinska Institutet
Stockholm, Sweden

#### I confirm that I am 18 years old or older and I consent to participating in this study. I understand that my answers are anonymous and will be saved and used for research purposes.*

( ) Yes

( ) No

#### I have a confirmed diagnosis of obsessive-compulsive disorder.*

( ) Yes

( ) No

### How old are you?*

_________________________________________________

#### What is your gender?*

( ) Female

( ) Male

( ) Other

( ) I prefer not to say

#### Country:*

#### What is the highest level of education you have completed? *

( ) Less than primary school

( ) Primary school completed

( ) Secondary school completed

( ) College/university completed

( ) Post-graduate degree

#### What is your current occupation?*

( ) Employed full-time

( ) Employed part-time

( ) Unemployed (able to work)

( ) Unemployed (unable to work)

( ) Retired

( ) Student

( ) Other. Please specify:: _________________________________________________

### If you remember, please indicate how old you were, approximately, when your OCD symptoms started (i.e., when they started to be time consuming [e.g., they were taking more than 1 hour a day] or started to significantly interfere with your functioning [e.g., it was difficult for you to follow a normal routine or to participate from occupational and social activities]):

_________________________________________________

#### Have you also been diagnosed with any of the following mental health conditions (select all that apply, if any):*

[ ] Body dysmorphic disorder

[ ] Hoarding disorder

[ ] Trichotillomania (hair-pulling disorder)

[ ] Excoriation (skin-picking) disorder

[ ] Autism spectrum disorder

[ ] Attention-deficit/hyperactivity disorder (ADHD)

[ ] Anxiety disorder (e.g., social anxiety disorder, panic disorder, generalized anxiety disorder)

[ ] Depression

[ ] Bipolar disorder

[ ] Psychotic disorder

[ ] Eating disorder

[ ] Substance use disorder

[ ] Other. Please specify:: _________________________________________________

[ ] I don't have any other mental health conditions, besides the OCD

## Treatment

#### Have you ever taken medication for your OCD?*

( ) Yes

( ) No

### If you remember, please indicate how old you were, approximately, when you received your first pharmacological treatment for OCD:

_________________________________________________

#### Are you currently taking medication for your OCD?*

( ) Yes

( ) No

#### What kind of medication are you taking for your OCD? (select all that apply)*

[ ] I take antidepressant medication for my OCD (e.g., Zoloft, Prozac, Celexa, Cipralex, Anafranil)

[ ] I take antipsychotic medication for my OCD (e.g., Abilify, Risperdal, Seroquel, Zyprexa)

[ ] I take other medications for my OCD. Please specify:: _________________________________________________

[ ] I don’t know

#### Have you ever received psychological treatment for your OCD?*

( ) Yes

( ) No

### If you remember, please indicate how old you were, approximately, when you received your first psychological treatment for OCD:

_________________________________________________

#### Are you currently receiving psychological treatment for your OCD?*

( ) Yes

( ) No

## Physical health

### What is your current weight, approximately? (please, indicate in the box what measure you are using; e.g., 70 kg, 154.3 lbs, 11 st)*

_________________________________________________

### What is your height, approximately? (please, indicate in the box what measure you are using; e.g., 170 cm, 5’7’’)*

_________________________________________________

#### Do you currently have any of the following health problems, confirmed by a doctor? (select all that apply, if any)*

[ ] Previous acute cardiovascular or cerebrovascular event (e.g., myocardial infarction, stroke)

[ ] Cardiovascular disorder (e.g., arrhythmia, venous thrombosis, heart failure)

[ ] High blood pressure (hypertension)

[ ] Type 2 diabetes

[ ] High cholesterol (e.g., dyslipidemia, hypercholesterolemia, hyperlipidemia)

[ ] Gastrointestinal conditions (e.g., gastroesophageal reflux, inflammatory bowel disease, irritable bowel syndrome)

[ ] Respiratory conditions (e.g., asthma, emphysema, bronchitis, pulmonary embolism)

[ ] Allergies (e.g., hay fever, allergic hives, food allergy)

[ ] Musculoskeletal conditions (e.g., arthrosis, osteoporosis, carpal tunnel, back pain)

[ ] Autoimmune diseases (e.g., rheumatoid arthritis, Hashimoto's thyroiditis, celiac disease, Graves' disease, type 1 diabetes)

[ ] Neurological diseases (e.g., epilepsy, Parkinson disease, multiple sclerosis)

[ ] Migraine headaches

[ ] Any type of cancer or tumor

[ ] Other. Please specify:: _________________________________________________

[ ] I don't have any health problems confirmed by a doctor

#### Are you currently on medication for any physical health condition? (select all that apply)*

[ ] No, I do not take medication for any physical health condition

[ ] Yes, I take medication for high blood pressure

[ ] Yes, I take medication for diabetes

[ ] Yes, I take medication for high cholesterol

[ ] Yes, I take medication for another physical health condition. Please specify:: _________________________________________________*

#### In general, how would you say your health is:*

( ) Excellent

( ) Very good

( ) Good

( ) Fair

( ) Poor

## Lifestyle habits

#### Do you smoke?*

( ) No

( ) Yes

### How many cigarettes per day, approximately?*

_________________________________________________

#### Have you smoked in the past?*

( ) No

( ) Yes, but I quit

### How old were you when you quit, approximately?*

_________________________________________________

#### Do you use snuff or other smokeless tobacco products (e.g., chewing tobacco)?*

( ) No

( ) Yes

### How many cans/boxes per week, approximately? (if applicable, otherwise please write 0)*

_________________________________________________

#### Have you used snuff/smokeless tobacco in the past?*

( ) No

( ) Yes, but I quit

### How old were you when you quit, approximately?*

_________________________________________________

#### How often do you have a drink containing alcohol?*

( ) Never

( ) Monthly or less

( ) 2-4 times a month

( ) 2-3 times a week

( ) 4 or more times a week

#### How many drinks containing alcohol do you have on a typical day when you are drinking?*

( ) 1 or 2

( ) 3 to 4

( ) 5 to 6

( ) 7 to 9

( ) 10 or more

#### How often do you have six or more drinks on one occasion?*

( ) Never

( ) Less than monthly

( ) Monthly

( ) Weekly

( ) Daily or almost daily

#### How often in the past year have you used an illegal drug (e.g., marijuana, cocaine, heroin, stimulants or hallucinogens) or used a prescription medication for non-medical reasons?*

( ) Never

( ) Less than monthly

( ) Monthly

( ) Weekly

( ) Daily or almost daily

#### During the last 7 days, on how many days did you do vigorous physical activities (for at least 10 minutes at a time) that made you breathe much harder than normal, like heavy lifting, digging, aerobics or fast bicycling?*

( ) I did not do any vigorous physical activity during the last 7 days

( ) 1-3 days per week

( ) 4-5 days

( ) 6-7 days

#### During the last 7 days, how much time did you usually spend doing vigorous physical activities on one of those days?*

( ) Less than 20 minutes per day

( ) 21-59 min per day

( ) 1 hour or more per day

( ) Don’t know/Not sure

#### During the last 7 days, on how many days did you do moderate physical activities (for at least 10 minutes at a time) that made you breathe somewhat harder than normal, like carrying light loads, bicycling at a regular pace or doubles tennis? Do not include walking.*

( ) I did not do any moderate physical activity during the last 7 days

( ) 1-3 days per week

( ) 4-5 days

( ) 6-7 days

#### How much time did you usually spend doing moderate physical activities on one of those days?*

( ) Less than 20 minutes per day

( ) 21-59 min per day

( ) 1 hour or more per day

( ) Don’t know/Not sure

#### During the last 7 days, on how many days did you walk for at least 10 minutes at a time?*

( ) I did not walk for at least 10 minutes during the last 7 days

( ) 1-3 days per week

( ) 4-5 days

( ) 6-7 days

#### How much time did you usually spend walking on one of those days?*

( ) Less than 20 minutes per day

( ) 21-59 min per day

( ) 1 hour or more per day

( ) Don’t know/Not sure

#### During the last 7 days, how much time did you spend sitting on a weekday?*

( ) 3 hours or less per day

( ) 4-7 hours per day

( ) 8 hours or more per day

( ) Don’t know/Not sure

#### How often do you eat vegetables? *

( ) Twice a day or more often

( ) Once a day

( ) A few times weekly

( ) Once a week or less

#### How often do you eat fruits or berries?*

( ) Twice a day or more often

( ) Once a day

( ) A few times weekly

( ) Once a week or less

#### How often do you eat fish or shellfish?*

( ) Three times a week or more often

( ) Twice a week

( ) Once a week

( ) A few times a month or less

#### How often do you eat sugary foods like sweets, chocolates, buns, soda, etc.?*

( ) Twice a day or more often

( ) Once a day

( ) A few times weekly

( ) Once a week or less

#### How often do you eat red meat or other meat products?*

( ) Twice a day or more often

( ) Once a day

( ) A few times weekly

( ) Once a week or less

#### How often do you eat processed food high in salt (e.g., packaged salty snacks, salty fast food, bacon)? *

( ) Twice a day or more often

( ) Once a day

( ) A few times weekly

( ) Once a week or less

#### How often do you eat breakfast?*

( ) Every morning

( ) Almost every morning

( ) A few times a week

( ) Once a week or less

#### How many hours do you sleep per night, on average?*

( ) 6 hours or less

( ) 7-9 hours

( ) 9 hours or more

#### Over the last two weeks, how often did you wake up feeling fresh and rested?*

( ) None of the time

( ) A little of the time

( ) Some of the time

( ) A good bit of the time

( ) Most of the time

( ) All of the time

#### Have you thought about changing your lifestyle habits (e.g., stop smoking if you smoke, increase physical activity, improve dietary habits)? *

( ) Yes

( ) No

#### What would you like to change? (select all that apply)*

[ ] Quit smoking

[ ] Drink less alcohol or quit drinking alcohol

[ ] Quit drug consumption

[ ] Increase physical activity

[ ] Eat healthier

[ ] Sleep better

[ ] Other. Please specify:: _________________________________________________

#### Have you previously tried to change your lifestyle habits?*

( ) Yes

( ) No

#### Have you been successful? *

( ) Yes

( ) No

### If you wish, please tell us what lifestyle habits you have managed to change:

_________________________________________________

### If you wish, please tell us why you think you were not successful at changing your lifestyle habits:

_________________________________________________

#### Why not? (select one main reason)*

( ) I am satisfied with my current lifestyle

( ) I don’t have time to incorporate changes to my lifestyle

( ) I believe it is going to be very difficult for me to make lifestyle changes because of my OCD

( ) I believe it is going to be very difficult for me to make lifestyle changes because of my other mental health problems

( ) I believe changing lifestyle habits would be too expensive

( ) Other. Please specify:: _________________________________________________*

#### Did your lifestyle habits change during the COVID-19 pandemic period?*

( ) No

( ) Yes, they improved. If you wish, please specify:: _________________________________________________

( ) Yes, they got worse. If you wish, please specify:: _________________________________________________

## Current OCD symptoms

### The following statements refer to experiences that many people have in their everyday lives. Choose the option that best describes **HOW MUCH that experience has DISTRESSED or BOTHERED you DURING THE PAST MONTH**.

#### I check things more often than necessary*

( ) Not at all

( ) A little

( ) Moderately

( ) A lot

( ) Extremely

#### I get upset if objects are not arranged properly*

( ) Not at all

( ) A little

( ) Moderately

( ) A lot

( ) Extremely

#### I find it difficult to touch an object when I know it has been touched by strangers or certain people*

( ) Not at all

( ) A little

( ) Moderately

( ) A lot

( ) Extremely

#### I find it difficult to control my own thoughts*

( ) Not at all

( ) A little

( ) Moderately

( ) A lot

( ) Extremely

#### I repeatedly check doors, windows, drawers, etc.*

( ) Not at all

( ) A little

( ) Moderately

( ) A lot

( ) Extremely

#### I get upset if others change the way I have arranged things*

( ) Not at all

( ) A little

( ) Moderately

( ) A lot

( ) Extremely

#### I sometimes have to wash or clean myself simply because I feel contaminated*

( ) Not at all

( ) A little

( ) Moderately

( ) A lot

( ) Extremely

#### I am upset by unpleasant thoughts that come into my mind against my will*

( ) Not at all

( ) A little

( ) Moderately

( ) A lot

( ) Extremely

#### I repeatedly check gas and water taps and light switches after turning them off*

( ) Not at all

( ) A little

( ) Moderately

( ) A lot

( ) Extremely

#### I need things to be arranged in a particular way*

( ) Not at all

( ) A little

( ) Moderately

( ) A lot

( ) Extremely

#### I wash my hands more often and longer than necessary*

( ) Not at all

( ) A little

( ) Moderately

( ) A lot

( ) Extremely

#### I frequently get nasty thoughts and have difficulty in getting rid of them*

( ) Not at all

( ) A little

( ) Moderately

( ) A lot

( ) Extremely

## A new lifestyle intervention for OCD

### A lifestyle intervention to promote healthier lifestyles in people with OCD is currently being developed. The intervention will include group educational sessions about healthy lifestyles and group exercise sessions to increase physical activity. We would like to know your views on the intervention and its format.

#### Do you think that a lifestyle intervention for OCD is a good initiative?*

( ) Yes

( ) No

### If you wish, please share your thoughts on why a lifestyle intervention for OCD is important:

____________________________________________

____________________________________________

____________________________________________

____________________________________________

#### Do you think that your health would benefit from an intervention to change lifestyle habits?*

( ) Yes

( ) No

#### If such intervention were offered to you, would you like to participate?*

( ) Yes

( ) No

#### Would you be prepared to carry a device to track your steps and activity during the day? *

( ) Yes

( ) No

#### Would you be prepared to join weekly exercise group sessions?*

( ) Yes

( ) No

#### Would you be prepared to send photos of your meals in order to track your diet? *

( ) Yes

( ) No

### If you wish, feel free to suggest components that you would like the lifestyle intervention to include:

____________________________________________

____________________________________________

____________________________________________

____________________________________________

## Thank you for taking part in our survey!

Your response is very important to us and will help us get a better understanding of the physical health and lifestyle habits of people with OCD.
